# Machine Learning Analysis of Immune Cells for Diagnosis and Prognosis of Cutaneous Melanoma

**DOI:** 10.1155/2022/7357637

**Published:** 2022-01-27

**Authors:** Huibin Du, Yan He, Wei Lu, Yu Han, Qi Wan

**Affiliations:** Department of Ophthalmology, People's Hospital of Leshan, Leshan, China

## Abstract

Tumor infiltration, known to associate with various cancer initiations and progressions, is a promising therapeutic target for aggressive cutaneous melanoma. Then, the relative infiltration of 24 kinds of immune cells in melanoma was assessed by a single sample gene set enrichment analysis (ssGSEA) program from a public database. The multiple machine learning algorithms were applied to evaluate the efficiency of immune cells in diagnosing and predicting the prognosis of melanoma. In comparison with the expression of immune cell in tumor and normal control, we built the immune diagnostic models in training dataset, which can accurately classify melanoma patients from normal (LR AUC = 0.965, RF AUC = 0.99, SVM AUC = 0.963, LASSO AUC = 0.964, and NNET AUC = 0.989). These diagnostic models were also validated in three outside datasets and suggested over 90% AUC to distinguish melanomas from normal patients. Moreover, we also developed a robust immune cell biomarker that could estimate the prognosis of melanoma. This biomarker was also further validated in internal and external datasets. Following that, we created a nomogram with a composition of risk score and clinical parameters, which had high accuracies in predicting survival over three and five years. The nomogram's decision curve revealed a bigger net benefit than the tumor stage. Furthermore, a risk score system was used to categorize melanoma patients into high- and low-risk subgroups. The high-risk group has a significantly lower life expectancy than the low-risk subgroup. Finally, we observed that complement, epithelial-mesenchymal transition, and inflammatory response were significantly activated in the high-risk group. Therefore, the findings provide new insights for understanding the tumor infiltration relevant to clinical applications as a diagnostic or prognostic biomarker for melanoma.

## 1. Introduction

Melanoma is the most aggressive type of cutaneous cancer derived from the melanocyte lineage, with the highest metastasis and mortality rate [1]. Despite melanoma contributing to only 5% of all skin-related cancers, it accounts for approximately 80% of deaths related to skin tumors. Like most other cancers, surgical enucleation and drug therapy are difficult to treat once it has metastasized [2–4]. In addition, it is difficult to detect early, and the majority of patients with melanoma were diagnosed at an advanced stage [5, 6]. Currently, cancer treatment guidance and prognosis prediction are largely determined by the TNM staging system. However, the clinical experience revealed that many patients, even within the same TNM stage, have differences in overall survival [7]. The clinical limitations of the TNM stage are increasingly becoming apparent. Thus, it is critical to identify novel biomarkers for early diagnosis and prognostic prediction.

Growing studies have recently reported that the tumor microenvironment plays a crucial role in the initiation and development of numerous malignant tumors [8, 9]. In the tumor microenvironment, the type, location, and function of immune cells are intimately associated with the clinical outcome [10, 11]. Tumor cells regarded as antigens will attract immune cells and leukocytes by many chemokines to influence the immune response. Moreover, the immune escape of a tumor cell was considered a crucial factor in tumorigenesis [12–14]. The prognostic value of the tumor microenvironment also had been demonstrated in numerous melanoma experiments. High immune infiltration in melanoma is also shown to be associated with a favorable prognosis. Currently, immune checkpoint inhibitors, such as CTLA-4, PD-1, and PD-L1 inhibitors, are pivotal therapies for the treatment of advanced melanoma [15, 16]. Therefore, it is promising to explore the tumor microenvironment-associated potential novel biomarkers for early diagnosis, prognosis prediction, and melanoma patient management. Besides tumor cells, melanoma also commonly includes various types of immune cells, which may be regarded as a potential diagnostic signature to classify tumors from suspected patients. Thus, systematically evaluated infiltrating immune cells were recognized as significant supplemental biomarkers to the TNM stage for diagnosis and prognosis prediction. Fortunately, the numerous transcriptome profiles deposited in the availability of public databases could provide immense data to investigate the infiltration of immune cells in melanoma.

Machine learning is a powerful tool to analyze and summarize complex datasets, which can provide various computational approaches to predict clinical diseases [17]. Previously, several algorithms have been successfully applied to diagnose and predict diseases, including logistic regression model [18], support vector machine [19], random forests analysis [20], and artificial neural network [21]. Compared to classical methods, machine learning often has remarkably high sensitivity and specificity. Also, machine learning is the best choice to process the increasingly growing genomic data and clinical information in oncology research and predict the susceptibility, survival, and recurrence of cancer.

Therefore, in this research, we, firstly, estimate the proportions of 24 immune cells in 944 samples (653 tumors and 291 normal controls) according to their gene expression at the mRNA level. Next, we use multicategory machine learning to identify several important immune cell signatures and construct diagnostic and prognostic models, which manifest important implications in terms of melanoma patient diagnosis and prognosis.

## 2. Materials and Methods

### 2.1. Melanoma Collection and Normal Controls

Melanoma patients were collected from public datasets. The eligible datasets were downloaded from the GEO database (https://www.ncbi.nlm.nih.gov/geo) and the UCSC Xena website (http://xena.ucsc.edu/public-hubs/). Finally, five datasets consisting of 944 samples, including TCGA, GSE3189, GSE15605, GSE46517, and GSE54467, were screened out for this study. *R* software was used to process the raw transcriptome profiles of RNA sequencing data. To begin, the probe IDs were annotated using platform annotation metadata. The median expression value will be generated to reflect the gene expression level for the same gene corresponding to multiple IDs. Then, genes with a variance of 0 will be eliminated for their low expression level. After that, log2 (*x* + 1) conversion was used to normalize the raw matrix data.

### 2.2. Estimation of Immune Cell Types

ssGSEA algorithm was applied to estimate the proportions of immune cells and transform the normalized gene expression data into 24 human immune cell types expression, which included the dendritic cells (DCs), immature DCs (iDC), activated DCs (aDC), plasmacytoid DCs (pDC), natural killer (NK) cells, CD56dim NK cells, CD56bright NK cells, Mast cells, macrophages, neutrophils, eosinophils, B cells, cytotoxic cells, and T cells. Also, the T cells were subdivided into the T central memory cells (Tcm), T effector memory cells (Tem), CD8 T cells, regulatory T cells (Treg), Tgd cells, T follicular helper cells (TFH), and T helper cells, namely Th1, Th2, and Th17 [22, 23].

### 2.3. Diagnostic Analysis

Firstly, these samples were categorized into tumor tissue groups and normal tissue groups. Then, different analyses of immune cells between the tumor and normal cells were carried out, and the *p* values less than 0.05 were regarded as the differently-expressed immune cells (DEICs). Then, we used the Upset plot to explore the overlap of DEICs among multiple datasets. To develop a diagnostic model with selected DEICs, five machine learning methods, including random forests (RF), logistic regression (LR), support vector machines (SVM), neural network (NNET), and least absolute shrinkage and selection operator (LASSO), combined with five-fold cross-validation, were systematically performed to construct the models in the TCGA dataset. The specificity and sensitivity of the diagnostic models were assessed by the receiver operating characteristic (ROC) curves. Principal component analysis was applied to determine whether these DEICs could definitely classify tumors from normal controls. The diagnostic model was also validated in another three independent datasets. Besides, the diagnostic score of each sample was calculated using the LR coefficients and corresponding expression level. The formula is ∑_*i*=1_^*N*^(coe*f*_*i*_ × exp  *r*_*i*_), which could well distinguish the normal and tumor tissues. Furthermore, immunohistochemistry was performed to compare the different infiltrating immune cells between melanoma and normal skin tissues. We estimated the immunohistochemical images by combining the percentage of positively stained cells with the staining intensity score.

### 2.4. Prognostic Analysis

To explore the most significant immune cells in the prognosis of melanoma, the qualified melanoma samples in TCGA were equally divided into training and testing samples at random. Multicategory machine learning methods, including LASSO, SVM- recursive feature elimination (SVM-RFE), and RF-feature selection (RF-FS), were conducted to identify the important immune cells in the training dataset. Then, the Cox regression method was performed to develop a prognostic model with selected immune cells. The risk scores were generated by the formula: ∑_*i*=1_^*N*^(coe*f*_*i*_ × exp  *r*_*i*_), where N stands for the number of cells, expr_i_ indicates the expression of cells, and coe*f*_*i*_ indicates the coefficient of cox regression. The patients in the training dataset were subsequently estimated by the risk formula, and then, the patients were divided into high- and low-risk groups based on the best cutoff risk score. Kaplan–Meier survival curves were created to assess the differences between the high- and low-risk groups, and log-rank tests were used to determine the significance. The predictive accuracy of the model for 5-year overall survival was estimated by the area under the curve (AUC) of receiver operating characteristic curves (ROC). Moreover, to demonstrate the result's robustness, the immune cell-related signature was further validated in the testing dataset and GSE54467. Finally, nomograms were constructed in this study based on patients' clinical characteristics and risk scores. To compare the prediction and actual survival, calibration curves were drawn. Decision curves were also plotted to discriminate the clinical usefulness of the nomogram and tumor stage.

### 2.5. Stratified Analysis

To investigate the connection between the risk score distribution and clinical features, the stratified analysis of clinical features, containing age, stage, gender, race, vital status, and tumor status, was conducted. Moreover, the univariate and multivariate cox regressions were carried out to evaluate the prognostic value of the risk score and clinical features. Next, to study the possible biological characteristics between the high- and low-risk groups, gene expression data correlated to immune checkpoint regulators and epithelial-mesenchymal transition (EMT) were investigated [24–27]. Firstly, the expression data of these genes were extracted, and then, they were classified into high- and low-risk groups using the optimal cutoff value. Then, stratified analyses of the corresponding genes were conducted.

### 2.6. Gene Set Enrichment Analysis

GSEA was performed to discover the significant pathways enriched in molecular mechanisms of Low- vs. high-risk groups using the “clusterProfiler” package in the *R* software. Firstly, all genes were produced by the “Limma” differential analysis of low- vs. high-risk groups and preranked using the log2 fold change of the expression values. Then, the cancer hallmark set (h.all.v7.0.symbols) and the KEGG set (c2.cp.kegg.v7.0.symbols) in GSEA were performed to explore the significant pathways associated with different groups of melanoma. The random sample permutations were 1000, and the *q* value <0.05 was the significance threshold.

### 2.7. Statistical Analysis

Every statistical analysis was executed using the *R* package (v.3.6.0) and corresponding packages. The Upset plot was drawn by the “UpSetR” package. The ssGSEA method was estimated by the “GSVA” package. LASSO and LR analysis were calculated by the “glmnet” package. SVM and SVM-RFE methods were conducted by the “e1017” package. RF and RF-FS algorithms were applied by “randomForest” and “varSelRF” packages, respectively. The NNET method was performed by the “nnet” package. The optimal cutoff value was generated by applying the “survminer” package. Kaplan–Meier survival curves and ROC curves were drawn by “survival” and “survivalROC” packages, respectively. GSEA analysis was performed by the “clusterProfiler” package. In all statistical tests, *p* < 0.05 was considered statistically significant.

## 3. Results

### 3.1. Melanoma Collection and Normal Controls

**Figure 1:**
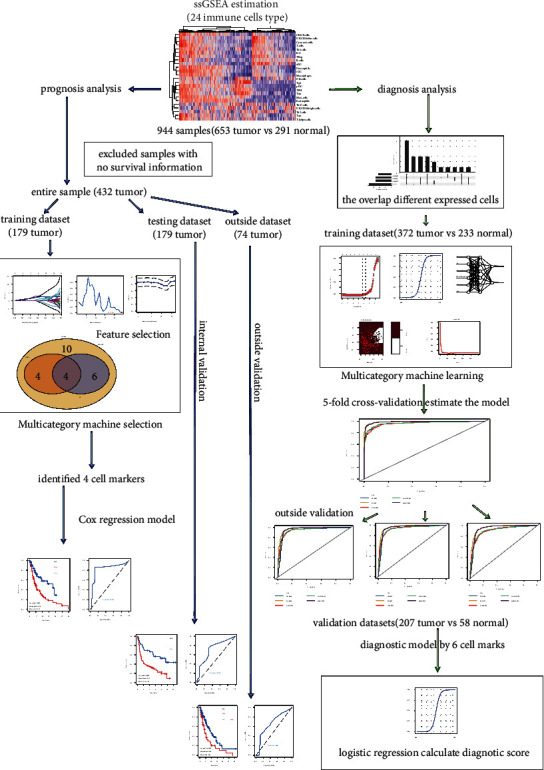
The complete workflow of the analysis in this study.

Totally, 944 samples were selected for the subsequent analysis, which were acquired from the five datasets, including the TCGA of melanoma, GSE3189, GSE15605, GSE46517, and GSE54467. The TCGA of melanoma was obtained from the UCSC Xena database, which included 372 melanomas and 233 healthy controls. GSE3189 contained 45 melanoma tumors and 25 normal controls. GSE15605 included 58 melanoma tumors and 16 normal healthy controls. GSE46517 contained 121 samples, which included 104 melanoma tumors and 17 normal controls. GSE54467 dataset had 74 melanoma samples alone with no healthy control. Moreover, 870 samples obtained from the TCGA of melanoma, GSE3189, GSE15605, and GSE46517 were used for diagnostic analysis. 446 melanoma samples obtained from the TCGA of melanoma and GSE54467 were conducted for prognostic analysis. The complete analysis workflow in this study is illustrated in Figure 1.

### 3.2. Differentially Expressed Immune Cells (DEICs)

**Figure 2:**
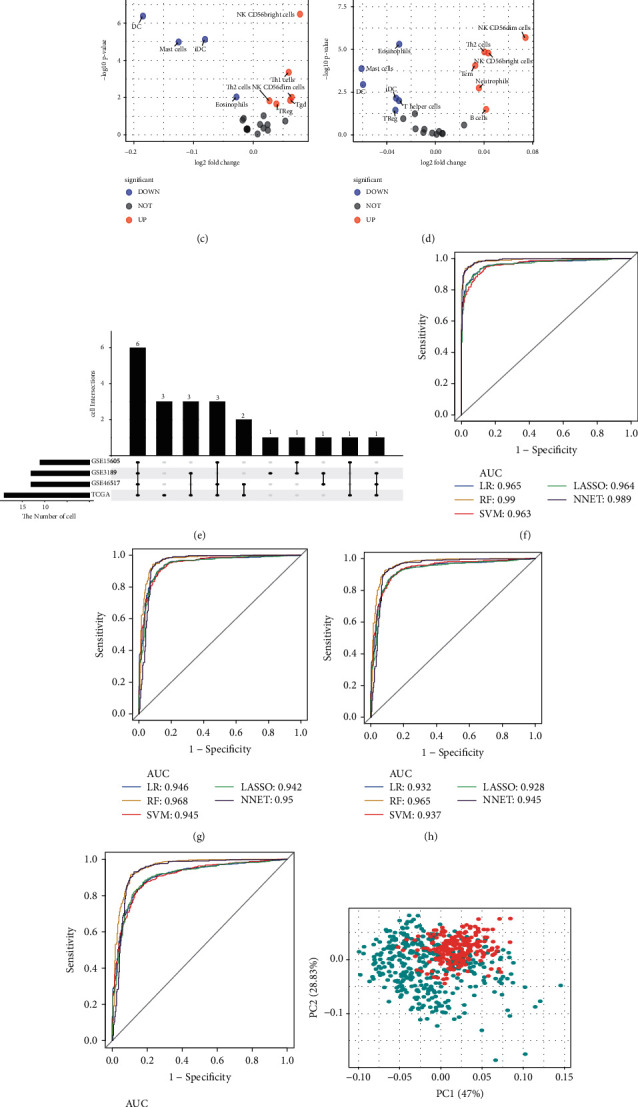
The immune cell-related diagnostic model of melanoma patients. (a–d) Volcano plots of TCGA dataset (a), GSE3189 (b), GSE15605 (c), and GSE46517 (d), which illustrated the differently-infiltrated immune cells between the melanoma and healthy controls. Red and blue plots mean statistical significance (*p* < 0.05). (e) The Upset plot of immune cells in different datasets. The amount of each dataset is shown by the dark bar on the left of the drawing. The black dots in the matrix on the right of the drawing indicate immune cell intersections. (f–i) Receiver operating characteristic (ROC) curves on multiple machine learning algorithms for the diagnostic model in TCGA dataset (f), GSE3189 (g), GSE15605 (h), and GSE46517 (i). AUC, area under ROC curve; LR, logistic regression; RF, random forests; SVM, support vector machines; LASSO, least absolute shrinkage and selection operator; NNET, neural network. (j) Principal component analysis of the expression of immune cells in the TCGA dataset. (k) Different distributions of diagnostic scores in multiple datasets. The box-violin plots indicate diagnostic scores at the median and interquartile range of value. ^*∗*^means *p* < 0.05; ^*∗∗*^ means *p* < 0.01; ^*∗∗∗*^means *p* < 0.001; ^*∗∗∗∗*^means *p* < 0.0001.

Firstly, the 24 immune cell expression matrix was calculated by ssGSEA. According to the standard of differential analysis, 19 DEICs were identified in TCGA, where 11 cells were highly infiltrated and 8 cells were lowly infiltrated (Figure 2(a)). 13 DEICs contained 4 highly infiltrated and 9 lowly infiltrated cells and were discovered in GSE3189 (Figure 2(b)). 11 DEICs were observed in GSE15605, which consisted of 6 highly infiltrated cells and 5 lowly infiltrated cells (Figure 2(c)). Besides, 7 highly infiltrated cells and 6 lowly infiltrated cells were identified in GSE46517 (Figure 2(d)). Eventually, 6 overlaps of DEICs were found in the four datasets (Figure 2(e)). These immune cells, including iDC, DC, Eosinophils, NK CD56bright cells, Mast cells, and Treg, were selected for subsequent research.

### 3.3. Constructing Diagnostic Model

**Figure 3:**
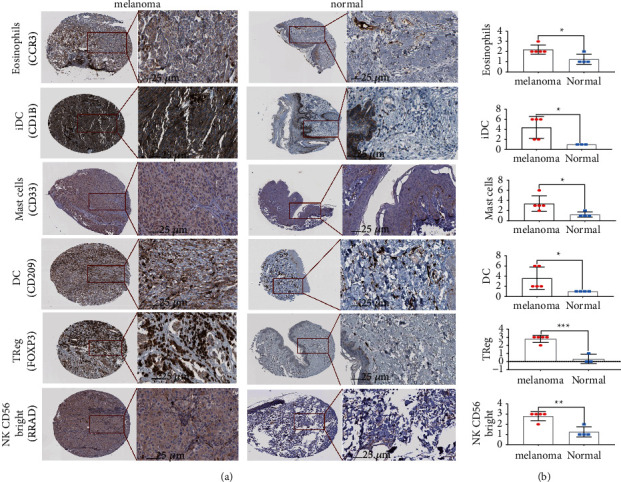
Immunohistochemical images. (a) Immunohistochemical images of six immune cells, including iDC, DC, eosinophils, NK CD56bright cells, Mast cells, and Treg, in the melanoma tissues and normal samples. (b) The box plots of the corresponding immunohistochemical scores (^*∗*^ represents *p* < 0.05, ^*∗∗*^ represents *p* < 0.01, and ^*∗∗∗*^ represents *p* < 0.001).

To construct a diagnostic model by applying the 6 identified DEICs and assess the effectiveness, five machine learning algorithms were comprehensively conducted to diagnose the melanoma samples from the normal healthy controls. Additionally, we also applied 5-fold cross-validation to estimate the accuracy of each model in the TCGA dataset. The ROC curves manifested that the six DEICs can accurately classify the melanoma patients from the normal ones (LR AUC = 0.965, RF AUC = 0.99, SVM AUC = 0.963, LASSO AUC = 0.964, and NNET AUC = 0.989) (Figure 2(f)). These diagnostic models were also validated in GSE3189, GSE15605, and GSE46517. Similarly, the ROC curves suggested over 90% AUC to identify melanomas from normal patients in all datasets (Figures 2(g)–2(i)). Principal components analysis illustrated that tumors and normal controls could be well distinguished according to the expression of six DEICs (Figure 2(j)). Next, we built a diagnostic score model with these DEICs using the LR method and used the diagnostic formula to calculate the score of each sample. The distributions of diagnostic scores in melanoma and healthy control were revealed in Figure 2(k). The violin plots manifested that the diagnostic values were differently distributed significantly in tumor samples and normal samples. To prove the results from the database, we used immunohistochemistry to confirm the infiltration of these immune cells. It also increased in melanoma compared to normal skin samples (Figures 3(a) and 3(b)).

### 3.4. Developing Prognostic Model

**Figure 4:**
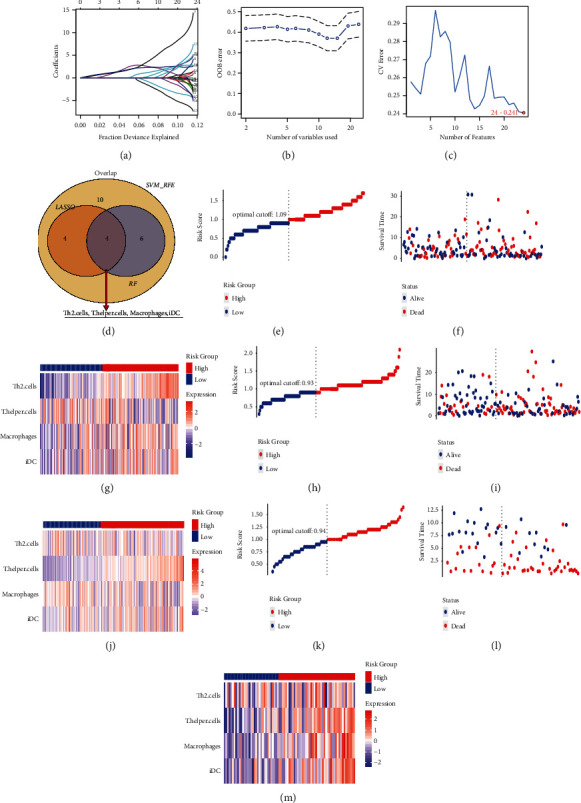
Prognostic feature selection and risk score model. (a) The coefficient profiles of the 24 immune cells in the LASSO algorithm. (b) Random forests feature selection (RF-FS) algorithm. The lowest point of the curve represents the lowest out-of-bag (OOB) error, which indicates the best immune cells-combined signature discovered by RF-FS. (c) Support vector machine-recursive feature elimination (SVM-RFE) algorithm. The highlighted point represents the lowest error rate, which indicates the best immune cells-combined signature identified by SVM-RFE. (d) The Venn plot of immune cells in RF-FS, SVM-RFE, and LASSO methods. E-G: the risk model in the training dataset: the risk scores distributions (e); overall survival time and vital status (f); the expression value of immune cells (g). (h–j)The risk model in the testing dataset: the risk scores distributions (h); overall survival time and vital status (i); the expression value of immune cells (j). (k–m) The risk model in the GSE54467 dataset: the risk scores distributions (k); overall survival time and vital status s (l); the expression value of immune cells (m).

**Figure 5:**
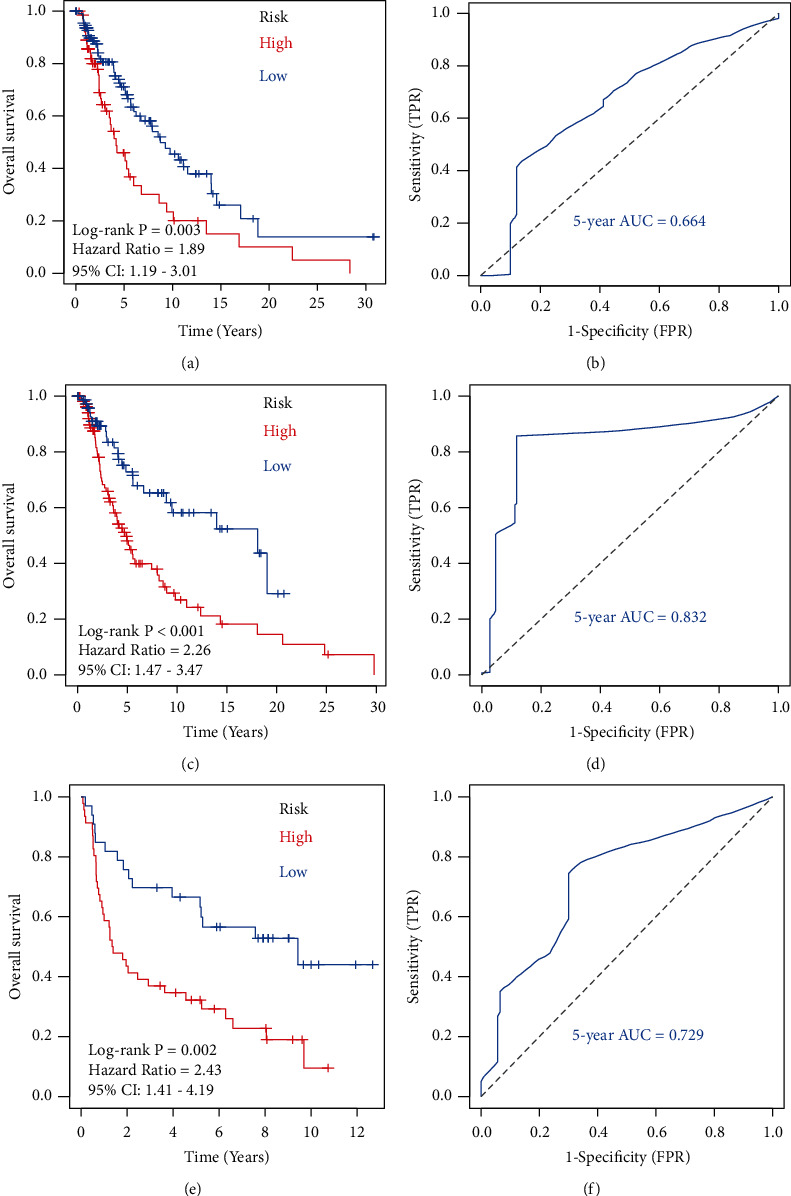
Construction and verification of immune cell biomarker for predicting survival. (a) The curves of Kaplan–Meier (KM) analysis for immune cells biomarker in the training dataset. (b) Receiver operating characteristic (ROC) analysis with area under the curve (AUC) for immune cell biomarker in 5 years at the training dataset. (c) KM analysis of immune cell biomarker in the testing dataset. (d) ROC curve with AUC value for immune cell biomarker in 5 years at the testing dataset. (e) KM analysis of immune cell biomarker in the GSE54467 dataset. (f) ROC curve with AUC value for immune cell biomarker in 5 years at the GSE54467 dataset.

After eliminating patients with no survival information, 432 melanoma samples were downloaded from the TCGA of melanoma and GSE54467. Firstly, the TCGA of the melanoma dataset was classified into training samples (*N* = 179) and testing samples at random (*N* = 179). The statistical results of the training and testing sample clinical information are displayed in Table 1, and no differences were observed between the two datasets. Next, combining the feature selection results of the LASSO method (Figure 4(a)), RF-FS method (Figure 4(b)), and SVM-RFE method (Figure 4(c)) showed that four overlapping immune cells were selected out (Figure 4(d)). Then, we used these immune cells in the training dataset to develop a risk score system by Cox regression. Next, the risk model rendered a risk score for each sample. The risk score distributions, overall survival (OS) time, vital status, and the corresponding expression of immune cells in the training (Figures 4(e)–4(g)), testing (Figures 4(h)–4(j)), and GSE54467 (Figures 4(k)–4(m)) datasets were respectively shown. Then, we used the optimal cut-off value to classify melanoma patients in the training dataset into high- or low-risk groups. The curves of Kaplan–Meier (KM) survival analysis manifested that the high-risk patient has a shorter survival time than a low-risk patient with a log-rank test *p*=0.003 (Figure 5(a)). The ROC curves manifested that the 5-year of AUC was 0.664 (Figure 5(b)). Moreover, to verify the robustness and applicability of the result, validation tests were performed in the testing set and the GSE54467 set. The testing and GSE54467 sets were classified into subrisk (high or low) groups accordingly. KM curves indicated that low-risk patients had significantly longer survival time than high-risk patients, regardless of being tested with log-rank *p* < 0.001 (Figure 5(c)) and GSE54467 with log-rank *p*=0.002 (Figure 5(e)). The 5-year of AUC in testing was 0.832 (Figure 5(d)) and the 5-year of AUC in GSE54467 was 0.729 (Figure 5(f)).

### 3.5. Nomogram Building and Validating

**Figure 6:**
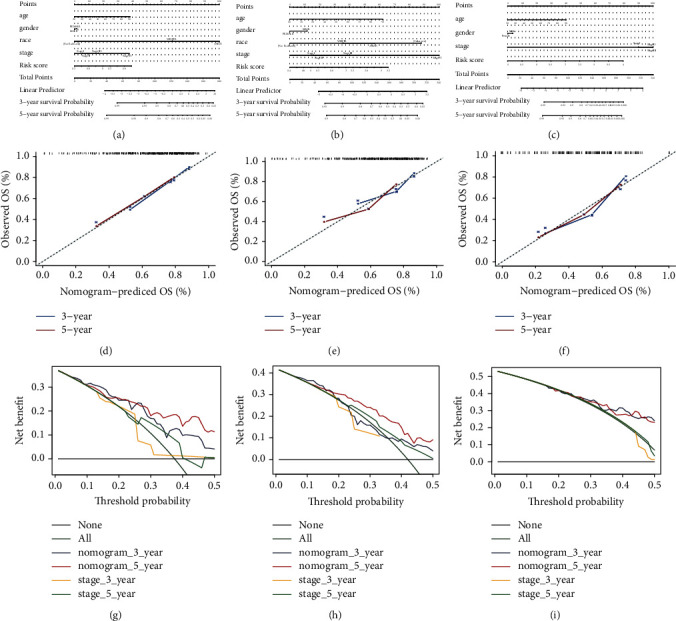
Construction and validation of nomogram. (a–c) Nomogram for predicting 3- and 5-year overall survival (OS) for melanoma patients in training (a), testing (b), and GSE54467 (c) based on the risk score and clinical characteristics. (d–f) Nomograms' calibration curves in terms of the correspondence between the predicted and observed 3 and 5 years of outcomes in training (d), testing (e), and GSE54467 (f). The 45° dashed line symbolizes perfect prediction, and the blue and red lines represent our nomogram's actual performance. (g–i) The decision curves for the nomogram, tumor stage at 3 and 5 years in training (g), testing (h), and GSE54467 (i).

To supply a simple and accurate method for OS prediction, the nomogram was built on the basis of clinical information and risk scores of patients in the training dataset (Figure 6(a)). The points of each parameter were then added to get a total point, which can predict the likelihood of OS at 3 and 5 years. In comparison to the ideal model, the calibration plots indicated that the nomogram worked well (Figure 6(d)). Moreover, similar nomograms were also constructed in the testing (Figure 6(b)) and GSE54467 (Figure 6(c)) datasets to prove the results. Surprisingly, the calibration plots in the testing (Figure 6(e)) and GSE54467 (Figure 6(f)) datasets for nomogram predicting 3- and 5-years' OS also worked well in comparison with the ideal model. Similarly, the nomograms' decision curve indicated that the nomogram model offered a bigger net benefit and a better clinical utility than the tumor stage, no matter in the training (Figure 6(g)), testing (Figure 6(h)), and GSE54467 (Figure 6(i)) datasets.

### 3.6. Relationships between Risk Model with Clinical Features and Gene Phenotypes

**Figure 7:**
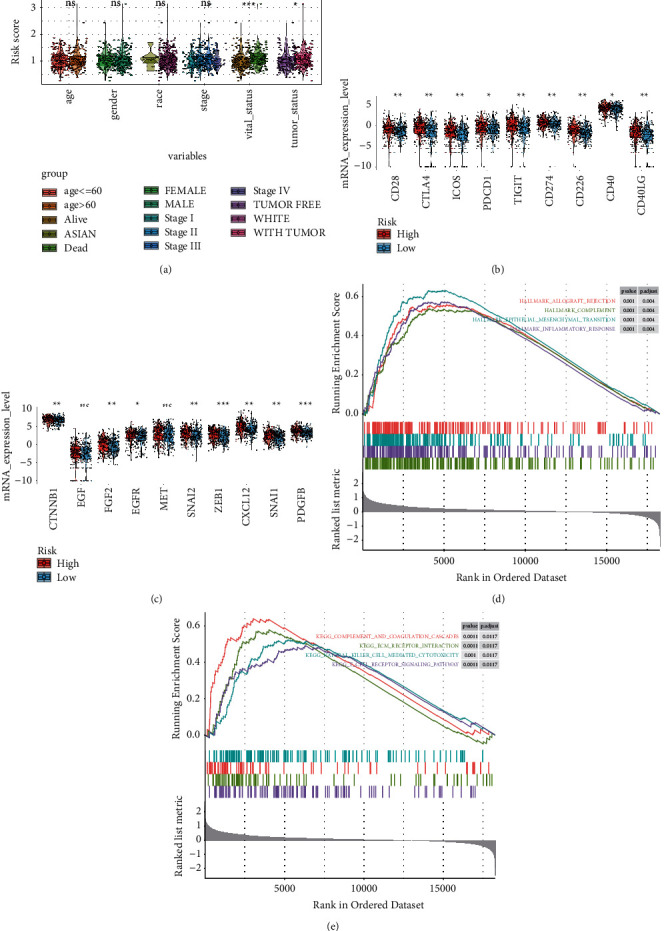
Stratified analysis and GSEA analysis. (a) The relationships between the distributions of the risk score and clinical variables, which contained age, race, gender, stage, vital status, and tumor status. (b) Box-violin plots of immune checkpoint-related genes between the high- and low-risk groups. (c) Box-violin plots of epithelial-mesenchymal transition (EMT)-related genes between the high- and low-risk groups. ^*∗*^ represents *p* < 0.05; ^*∗∗*^ represents *p* < 0.01; ^*∗∗∗*^ represents *p* < 0.001; ^*∗∗∗∗*^ represents *p* < 0.0001. (d, e) GSEA of high- vs. low-risk score groups using the gene sets of the cancer hallmark pathway (h.all.v7.0.symbols) (d) and KEGG pathway (c2.cp.kegg.v7.0.symbols) (e).

The associations between the risk model and clinical features were explored, and the violin plot manifested that the risk score only associated with the vital status and tumor status (Figure 7(a)). Other clinical characteristics, such as age, gender, race, and stage, had no effect on the risk score. Furthermore, univariate and multivariate Cox regressions were used to compare the prognostic value of the risk score and clinical features in training, testing, and GSE54467 datasets (Table 2). The univariate Cox analysis revealed that age, tumor status, stage, and risk sore were significantly associated with overall survival, however, the multivariate Cox analysis indicated that only the risk score was associated with OS significantly and could be considered an independent risk factor in training (HR = 3.517, *p*=0.005), testing (HR = 1.869, *p*=0.042), and GSE54467 datasets (HR = 2.661, *p* < 0.000). To investigate the correlations between the risk model and selected immune checkpoint-related genes, the subgroup analysis of immune checkpoint-related genes was performed. The violin plot revealed that CD28, CTLA4, ICOS, PDCD1, TIGIT, CD274, CD226, CD40, and CD40LG in the high-risk group had a higher expression value than those in the low-risk group (Figure 7(b)). Interestingly, the subgroup analysis of EMT-related genes showed that a majority of the EMT-related genes were differently expressed between the high- and low-risk groups. The genes expression levels of CTNNB1, FGF2, EGFR, SNAI2, ZEB1, CXCL12, SNAI1, and PDGFB were significantly higher in the high-risk group (Figure 7(c)).

### 3.7. Gene Set Enrichment Analysis

According to the selection standard, multiple significant cancer hallmark pathways were enriched, such as allograft rejection, complement, EMT, and inflammatory response (Figure 7(d)). Additionally, KEGG enrichment showed that complement and coagulation cascades, natural killer cell-mediated cytotoxicity, ECM receptor interaction, and T cell receptor signaling were positively active in the high-risk group (Figure 7(e)).

## 4. Discussion

In recent years, melanoma patients are becoming younger, with more advanced metastasis and a higher risk of death. Despite numerous advanced therapeutic methods being used to treat melanoma, such as chemotherapy, radiotherapy, and immunotherapies, their survival rate remains low [1, 3]. Besides, the traditional classification is often ineffective and lacks clinical benefits. Therefore, researchers are struggling to explore the new biomarkers to better diagnose and predict prognosis. Huang et al. identified eight immune-related gene biomarkers that could predict the prognosis of melanoma [28]. An RNA sequencing-based 12-gene signature was established by applying univariate and multivariate regression models to predict the prognosis of melanoma patients [29]. Lu et al. discovered a five-miRNA signature by analyzing the microarray dataset in the GEO database, which could be regarded as an independent prognostic biomarker in melanoma patients [30]. Recently, the tumor immune microenvironment in melanoma has become a research hotspot and is under active investigation [31]. Moreover, the immune cell types differentially distributed in the tumor tissue on diagnosis have attracted great interest in recent years. Therefore, in this study, we systematically analyzed the immune microenvironment and tried to establish a more evaluable and precise signature for advanced melanoma patients.

Various differential expressions of genes were recently analyzed to diagnose tumors. Nevertheless, little research attention looked at the effects of the immune cell on the diagnosis of melanoma. Firstly, we conducted the ssGSEA method to assess the relative expression of 24 kinds of human immune cells. Compared to normal tissues, the distribution of the immune cell was significantly higher in the tumor tissues. The overlapping DEICs were identified and put into machine learning analysis. The high sensitivity and specificity of multiple machine learning algorithms indicated that DECI was an efficient indicator for the diagnosis of melanoma. In addition, we built a diagnostic score model by logistic regression method, which could effectively distinguish the melanomas from the normal controls, replying that the immune system is closely associated with the tumorigenesis of melanoma. Similar results have been reported that the infiltration of the immune cell can be used to diagnose colon cancer, even all digestive system cancers [32, 33]. In this sense, immune infiltration opened a novel strategy for diagnosing and treating melanoma.

To subsequently investigate the prognostic value of the immune infiltration in melanoma, LASSO, RF-FS, and SVM-RFE methods were jointly applied to select the potential immune cells for building the prognostic model. Finally, four types of immune cells, including Th2 cells, T helper cells, Macrophages, and iDC, were employed to develop the risk model by the Cox regression method, which was also validated in the internal and external datasets. Among these immune cells, some have been proven to be associated with melanoma. For instance, approximately 70% of melanoma metastatic lymph nodes were detected in the distribution of immature DCs, which may take an immunosuppressive function in melanoma [34]. The Th1 and Th2 cells are in a somewhat balanced condition in the normal immunological milieu. The imbalance of Th1/Th2 is referred to as the Th2 bias, which severed the inhibitory effect on Th1 responses [35]. One of the mechanisms of tumor immune escape is the Th2 bias. Studies have demonstrated that the dominance of the Th2 cells could regulate chronic inflammation, which led to the metastasis of melanoma. Moreover, Falleni et al. proved that macrophage accumulation was a poor predictor of melanoma in a patient and might be considered a possible therapeutic target [36]. To assess the accuracy of prognostic prediction, we also built a nomogram-integrated risk score and clinical information. The calibration curve for the 3 and 5 years of outcomes showed that the nomogram worked well compared with the ideal model. Besides, in comparison with the tumor stage, the decision curve plots depicted that the nomogram model can acquire more benefit. The multivariate cox analysis also suggested that the risk score of immune cells-related biomarkers might be considered an independent prognostic factor in melanoma.

Based on the optimal cutoff of risk score, melanomas were classified into subrisk groups. The KM curves revealed that high-risk patients have a poor prognosis. Thus, to explore the underlying mechanism with different subgroups, the stratified analyses of clinical characteristics and gene phenotypes were performed. The risk score distribution of clinical features showed that the risk score was only correlated to vital status and tumor status. Presently, checkpoint blockade immunotherapies represent a promising strategy for cancer therapy and acquired extensive investigations [37, 38]. However, the efficacy of immunotherapies is dramatically varied in individual patients and different subtypes of cancer. In our research, the expression of immune checkpoint-related genes, including CD28, CTLA4, ICOS, PDCD1, TIGIT, CD274, CD226, CD40, and CD40LG, were highly expressed in high-risk patients. Besides, epithelial-mesenchymal transition (EMT) recognized the indictor for the invasion and progression of many cancers [39, 40]. The selected EMT-related genes are also included in our research, and the results also manifested that most of them are highly expressed in the high-risk group. Therefore, we have reason to suspect that our immune cell-related biomarker is linked to melanoma prognosis.

To further investigate the potential biological mechanism in the high-risk phenotype, the GSEA method was applied to analyze the candidate pathways. The results showed that the high-risk phenotype was positively associated with cancer hallmarks, such as allograft rejection, complement, EMT, and inflammatory response, which supported the previous findings that EMT and immune-associated genes were highly expressed in the high-risk group. The complement system, an essential constituent of innate immunity, affects tumor growth and metastasis by regulating chronic inflammation. Moreover, the KEGG pathway analysis showed that complement and coagulation cascades, ECM receptor interaction, natural killer cell-mediated cytotoxicity, and T cell receptor signaling pathways were enriched in the high-risk phenotype, which largely consisted with cancer hallmark analysis. The ECM-receptor interaction pathway is crucial in the metastasis of the tumor [41]. The importance of the ECM-receptor interaction pathway revealed that the tumor cells and the environment have a dynamic interaction [42].

## 5. Conclusion

To sum up, our study discovered several differential immune cells and proved the efficiency of immune cells in diagnosing and predicting the prognosis of melanoma. In clinical application and management, the developed diagnosis and prognosis models may give an easier and more accurate prediction for melanoma patients. However, our experiments are limited to bioinformatic analysis, and further experiments should be performed *in vitro* and *in vivo*.

## Figures and Tables

**Table 1:** Clinical characteristics of the train and test dataset. IQR: interquartile range; OS: overall survival.

	Level	Test samples	Train samples	*p*
*n*		179	179	
Age (median [IQR])		58.00 [47.00, 71.00]	58.00 [48.00, 70.00]	0.685
Gender (%)	Female	59 (33.0)	73 (40.8)	0.154
	Male	120 (67.0)	106 (59.2)	
Race (%)	(Not evaluated)	4 (2.2)	1 (0.6)	0.512
	(Unknown)	1 (0.6)	2 (1.1)	
	Asian	4 (2.2)	3 (1.7)	
	White	170 (95.0)	173 (96.6)	
Stage (%)		16 (8.9)	10 (5.6)	0.594
	I/II NOS	4 (2.2)	4 (2.2)	
	Stage I	32 (17.9)	35 (19.6)	
	Stage II	51 (28.5)	64 (35.8)	
	Stage III	67 (37.4)	58 (32.4)	
	Stage IV	9 (5.0)	8 (4.5)	
Vital_status (%)	Alive	94 (52.5)	94 (52.5)	1.000
	Dead	85 (47.5)	85 (47.5)	
Tumor_status (%)		2 (1.1)	4 (2.2)	0.452
	Tumor-free	76 (42.5)	84 (46.9)	
	With tumor	101 (56.4)	91 (50.8)	
OS time (median [IQR])		3.16 [1.51, 6.56]	3.47 [1.39, 6.68]	0.831

**Table 2:** Univariate and multivariate Cox regression of risk scores and clinical features correlated with the overall survival time in train, test, and GSE54467 datasets. IQR: interquartile range. HR: hazard ratio.

Train sample (*n* = 179)	Univariate analysis				Multivariate analysis			
Marker	*p* value	HR	Lower 0.95	Upper 0.95	*p* value	HR	Lower 0.95	Upper 0.95
Age	0.000	1.028	1.013	1.044	0.004	1.024	1.007	1.041
Gender	0.880	0.965	0.608	1.533	0.840	1.055	0.631	1.763
Race	0.002	0.157	0.047	0.521	0.074	0.329	0.097	1.114
Stage	0.004	1.493	1.140	1.957	0.009	1.466	1.099	1.957
Tumor_status	0.000	8.711	3.789	20.027	0.000	7.067	3.039	16.435
Risk score	0.005	2.619	1.333	5.147	0.005	3.517	1.457	8.492
Test sample (*n* = 179)								
Age	0.002	1.023	1.008	1.038	0.020	1.020	1.003	1.037
Gender	0.187	1.367	0.859	2.175	0.570	1.160	0.695	1.935
Race	0.474	0.484	0.066	3.531	0.015	0.063	0.007	0.585
Stage	0.012	1.471	1.088	1.988	0.000	1.909	1.387	2.626
Tumor_status	0.000	9.188	4.233	19.946	0.000	19.431	6.995	53.973
Risk score	0.018	2.028	1.131	3.637	0.042	1.869	1.022	3.417
GSE54467 sample (*n* = 79)								
Age	0.014	1.023	1.005	1.041	0.003	1.029	1.010	1.049
Gender	0.998	1.001	0.568	1.764	0.572	1.181	0.663	2.105
Stage	0.675	1.077	0.762	1.522	0.148	1.321	0.906	1.927
Risk score	0.000	2.683	1.670	4.312	0.000	2.661	1.654	4.281

## Data Availability

The datasets generated for this study can be found in the GEO database (GSE3189, GSE15605, GSE46517, and GSE54467; https://www.ncbi.nlm.nih.gov/geo/) and the UCSC Xena website (TCGA-SKCM; https://gdc.xenahubs.net).
